# The 2019 Conference on Health and Active Transportation: Research Needs and Opportunities

**DOI:** 10.3390/ijerph182211842

**Published:** 2021-11-11

**Authors:** David Berrigan, Andrew L. Dannenberg, Michelle Lee, Kelly Rodgers, Janet R. Wojcik, Behram Wali, Calvin P. Tribby, Ralph Buehler, James F. Sallis, Jennifer D. Roberts, Ann Steedly, Binbin Peng, Yochai Eisenberg, Daniel A. Rodriguez

**Affiliations:** 1 National Cancer Institute, National Institutes of Health, Bethesda, MD 20892, USA; 2 Department of Environmental and Occupational Health Sciences, School of Public Health, University of Washington, Seattle, WA 98195, USA; adannen@uw.edu; 3 Department of Urban Design and Planning, College of Built Environments, University of Washington, Seattle, WA 98195, USA; 4 Resource Systems Group (RSG) Inc., Department of Travel Behavior Research, Merritt Island, FL 32953, USA; michelle.lee@rsginc.com; 5 Nohad A. Toulan School of Urban Studies and Planning, College of Urban and Public Affairs, Portland State University, Portland, OR 97201, USA; krodge2@pdx.edu; 6 Department of Physical Education, Sport and Human Performance, Winthrop University, Rock Hill, SC 29733, USA; wojcikj@winthrop.edu; 7 Urban Design 4 Health, Inc., Rochester, NY 14620, USA; bwali@ud4h.com; 8 Department of Geography, University of Hong Kong, Hong Kong, China; cptribby@gmail.com; 9 Urban Affairs and Planning, Virginia Tech, Arlington, VA 22203, USA; ralphbu@vt.edu; 10 Herbert Wertheim School of Public Health and Human Longevity Science, University of California San Diego, La Jolla, CA 92093, USA; jsallis@ucsd.edu; 11 Mary MacKillop Institute of Health Research, Australian Catholic University, Melbourne 3000, Australia; 12 Department of Kinesiology, School of Public Health, University of Maryland, College Park, MD 20742, USA; jenrob@umd.edu; 13 Planning Communities LLC, Raleigh, NC 27615, USA; asteedly@planningcommunities.com; 14 National Center for Smart Growth, University of Maryland, College Park, MD 20742, USA; binbinpeng1991@gmail.com; 15 Department of Disability and Human Development, University of Illinois at Chicago, Chicago, IL 60608, USA; yeisen2@uic.edu; 16 Department of City & Regional Planning, College of Environmental Design, University of California-Berkeley, Berkeley, CA 94720, USA; danrod@berkeley.edu

**Keywords:** active transportation, COVID-19, climate change, physical activity, built environment, public health

## Abstract

Active transportation (AT) is widely viewed as an important target for increasing participation in aerobic physical activity and improving health, while simultaneously addressing pollution and climate change through reductions in motor vehicular emissions. In recent years, progress in increasing AT has stalled in some countries and, furthermore, the coronavirus (COVID-19) pandemic has created new AT opportunities while also exposing the barriers and health inequities related to AT for some populations. This paper describes the results of the December 2019 Conference on Health and Active Transportation (CHAT) which brought together leaders from the transportation and health disciplines. Attendees charted a course for the future around three themes: Reflecting on Innovative Practices, Building Strategic Institutional Relationships, and Identifying Research Needs and Opportunities. This paper focuses on conclusions of the Research Needs and Opportunities theme. We present a conceptual model derived from the conference sessions that considers how economic and systems analysis, evaluation of emerging technologies and policies, efforts to address inclusivity, disparities and equity along with renewed attention to messaging and communication could contribute to overcoming barriers to development and use of AT infrastructure. Specific research gaps concerning these themes are presented. We further discuss the relevance of these themes considering the pandemic. Renewed efforts at research, dissemination and implementation are needed to achieve the potential health and environmental benefits of AT and to preserve positive changes associated with the pandemic while mitigating negative ones.

## 1. Introduction

Active transportation (AT) is an important target for increasing physical activity and improving health while addressing environmental problems through reductions in vehicular emissions [1,2,3]. AT, defined as any self-propelled human-powered movement from one destination to another such as walking, cycling and use of wheelchairs, is strongly associated with land use characteristics and use of public transportation [4,5]. Additionally, skateboards, scooters and new mobility platforms such as e-bikes and e-scooters play a role in transportation and provide both opportunities and challenges to increasing physical activity [6]. AT contrasts with leisure-time activities, which may involve the same kind of physical activity, but do not necessarily involve destinations, a distinguishing feature of transportation. It can be difficult to categorize transportation activities with multiple functions such as a pleasant stroll to a café or a walk that combines exercise and a visit to the post office. Such ambiguities can be addressed via sensitivity analyses, such as repeated analyses using alternative categorization schemes. Despite the consensus concerning health and environmental benefits of AT among many health and transportation researchers, progress in increasing AT at the population level has slowed or halted in the United States; however, there are increases in AT for certain localities and demographic groups [7,8,9]. Between 2010 and 2018, pedestrian and cycling fatality rates fell or remained stable in several European countries as well as worldwide but they increased in the US and some other countries [10,11].

Data also reveal the dangers of AT for some people of color including African Americans and Hispanics [12], with higher pedestrian death rates for Hispanic and African American people and greater increases in mortality rates for African Americans. From 2009 to 2018, the age-adjusted pedestrian death rate in the U.S. increased from 1.4 to 1.8 per 100,000 for White pedestrians and 2.4 to 2.9 per 100,000 for Hispanics. The increase was 2.5 to 3.6 per 100,000 for African Americans, nearly double the increase seen for White and Hispanic pedestrians [13]. In the US, minority populations are more likely to reside in urban areas where traffic volumes are higher. Recent results suggest that racial bias and implicit prejudice are likely to play a major role in this disparity. For example, studies have revealed that motorists were twice as likely to not yield for African American pedestrians and that these pedestrians experienced wait periods in crosswalks that were one-longer than for White pedestrians [14,15,16].

As the coronavirus (COVID-19) pandemic progressed, new AT opportunities were created even though the barriers and health inequities related to AT for some populations were further exposed. For example, during periods of lockdown, communities closed roadways to motor vehicles, built pop-up bike lanes, and there was an overall increase in cycling in the United States as well as in Canada and countries throughout Europe—but often for leisure purposes [17]. It is unknown to what extent these environmental changes complied with accessibility design guidelines [18] in the same way permanent infrastructure does. A higher proportion of essential and frontline workers, who are also disproportionately low-income persons of color, used public transportation, mainly buses, throughout the pandemic [19]. Such use of public transportation is associated with increased AT via walking, wheelchair use or bicycling to access transit and then reach the users’ final destinations. For many of these public transit riders, their risk of COVID-19 exposure was potentially increased, particularly when transit service was cut so vehicle crowding remained high, or when journeys were lengthened due to reduction or suspension of service [20]. Secular trends, the pandemic, climate change, the continuing obesity epidemic in the US and pervasive disparities in safety and access suggest an ongoing need for research to increase AT while avoiding negative outcomes such as exposure to pathogens, increased risk of injury, or inadvertently reducing access for some.

The Transportation Research Board (TRB), Centers for Disease Control and Prevention (CDC), National Institutes of Health (NIH) and American College of Sports Medicine (ACSM) convened the Conference on Health and Active Transportation (CHAT) in December 2019 [21]. The CHAT focused on three themes, Innovative Practices, Building Strategic Institutional Relationships, and Identifying Research Needs and Opportunities [21]. In this paper we summarize the research needs and opportunities articulated in this conference and relate these results to two emerging challenges associated with the COVID-19 pandemic. The first is how to address the challenge of restoring public transportation use after the pandemic and the dramatic declines in transit use, transportation walking [22,23] and active commuting observed worldwide [24,25]. The second is how can we preserve and increase positive changes associated with the pandemic including increases in open streets and the boom in cycling. Although note much of the increase in cycling may be associated with leisure [17].

We argue that new approaches to AT research and implementation are required to achieve greater levels of AT in some countries and reverse the pandemic-related declines in transit use. More research demonstrating that AT has health benefits appears unlikely to result in increased AT, in part because transportation agencies are not required to consider all the health impacts of projects. Additional documentation of disparities in safety, infrastructure and access alone will not increase AT or reduce disparities. Further dissemination of research results concerning AT and health to health departments and promoting communication between the health and transportation sectors [26] may encourage partnerships, but fundamentally redesigned street infrastructure is needed to better accommodate a comprehensive and safe network for AT and to increase population AT levels. As discussed below, better incorporating financial and environmental benefits of AT infrastructure into transportation planning could accelerate progress towards better infrastructure. Such infrastructure needs to be developed equitably and with attention to context. 

Research will be needed to determine how such investments influence use and to inform successful strategies to accelerate development of infrastructure that encourages AT. The conference highlighted the need for application of a broader set of approaches, including deeper examination of equity considerations in AT planning and project implementation; employment of implementation science frameworks to better understand the components of transportation project delivery that results in health benefits; and the use of interdisciplinary approaches to study the economic, organizational and political dimensions of land use and infrastructure change. Rigorous evaluation of alternative policies, programs and infrastructure can better inform policy makers. Greater attention to evaluating emerging technologies and practices as well as increased attention to messaging and dissemination will be required to select and promote novel and effective efforts to advance AT. The conference also emphasized the need to engage community members and understand their needs and values related to transportation, neighborhoods and the social and built environment.

The CHAT focused on health and AT in the United States. The results of this work are relevant to diverse countries experiencing public health and environmental challenges related to lack of physical activity, obesity and the effects of automobile dependence on land use, public safety and air pollution [27,28]. We recognize that different settings and national circumstances will require distinct approaches and innovations, and we hope this paper and the materials discussed will stimulate further efforts to address AT and health worldwide [29,30,31]. Perhaps conferences modeled on the CHAT could be held in multiple countries or regions. Such conferences, including both transportation and health professionals, could help focus attention on context specific factors influencing positive and negative aspects of AT as well as develop strategies to encourage AT based on locally prioritized approaches.

## 2. The Conference on Health and Active Transportation (CHAT) and Its Context

The CHAT was held 11–12 December 2019, in Washington, D.C. [21]. This open meeting attracted 145 attendees included representatives of the transportation (50%) and public health (40%) sectors, with participants from federal, state, and local levels of government, private sector, national associations, nonprofit organizations, and metropolitan planning organizations (MPOs) as well as leading university researchers. This conference was designed as a follow-up to a 2015 conference: Moving Active Transportation to Higher Ground: Opportunities for Accelerating the Assessment of Health Impacts [32]. The CHAT included plenary sessions, research presentations and breakout groups in each of the three themes, designed identify research gaps that were refined via a combination of discussion and dot or multi-voting [33,34].

The CHAT occurred in the context of several efforts aimed at strengthening research activities, collaboration, practice and communication between the transportation and health sectors. Some of these efforts arose from the TRB Subcommittee on Health and Transportation (https://sites.google.com/site/trbhealthandtransport/ (accessed on 9 November 2021). The subcommittee has recently been elevated to full Committee status as the Committee on Transportation and Public Health in TRB [35]. Other recent collaborative efforts are associated with distinct aspects of the Transportation sector, including the National Cooperative Highway Research Program (NCHRP) and the American Association of State Highway and Transportation Officials (AASHTO) Committee on Environment and Sustainability. Overlap amongst participants in these distinct efforts and informal conversations reduced duplication and led to highly complementary products (Table 1).

We trace much of this work to the formation of the TRB subcommittee on Health and Transportation in 2011, summarized in a 2019 TRB centennial paper [41]. The subcommittee participated in the 2015 Moving to Higher Ground conference and an associated theme issue of TRNEWS [44]. The theme issue focused on health impacts of transportation practices for the broad transportation community of practitioners and planners as well as researchers, making a strong case that attention to health outcomes could complement traditional foci of the transportation sector on measures related to vehicular movement. A second major effort arising from the subcommittee was the formation of the Task Force on Arterials and Public Health (ADD55T). Arterial roadways were deemed a key target for incorporating health perspectives into optimizing transportation for communities, and the task force developed an extensive list of research ideas disseminated in a TRB circular [37] and in venues reaching both transportation [38] and public health [36] communities. Lastly, as a follow-up to the 2015 conference, the subcommittee organized the 2019 CHAT.

Two complementary efforts, also cited in Table 1, arose from other elements of the US Transportation sector. First an NCHRP research roadmap concerning transportation and public health was released in 2019 [42,43]. The roadmap was based on literature review and key informant interviews and identified 44 research gaps and 122 research needs, some of which involve AT, especially calls for better data and measurement of travel behavior and more evaluation research on the health and behavioral implications of transportation projects and policy. Second, a communications guide aimed at facilitating interchange between the transportation and health communities was released in 2019 [26]. This guide is an invaluable effort to enhance communication between transportation and health professionals. Finally, we note the NASEM 2019 report on improving physical activity surveillance in the United States that included a group of experts from the health and transportation sector, several of whom were involved in the CHAT and other activities mentioned above. It includes extensive recommendations related to surveillance of community supports for AT [39].

The CHAT research recommendations echo in part past themes in discussions of research needs at the intersection of transportation and health. For example, Sallis et al., (2004) emphasized needs for expanded conceptual models, activity-based travel measures, environmental justice, and relationships between exposure to air pollution and travel modes [1]. Litman (2013) discussed the need for diverse modelling approaches addressing policy and planning outcomes, empirical studies linking travel activity to safety, pollution exposure, access and physical activity and building support for policy reform aimed at improving transportation-related health outcomes [45]. These topics still demand attention.

In 2015, the Moving Active Transportation to Higher Ground Conference, which inspired the CHAT, touched on many of the same themes, and particularly emphasized data and modelling needs required to accelerate progress in addressing questions related to transportation and health. Adequate data and linkage between transportation and health data remain barriers to rigorous evaluation of health impacts of transportation infrastructure, programs and policies [32]. These overarching themes were further developed in the recent Research Roadmap for transportation and health [42]. Despite some overlap in contents, the resources described here, and past literature reviews and commentaries serve distinct functions and emphasized different aspects of the intersections between transportation and health.

The 2015 Moving Active Transportation to Higher Ground Conference also emphasized better incorporating health measurements and impact assessment into transportation practice. The arterials task force focused on a specific but critical setting in urban and suburban areas. The Communications guidebook [26] plays a vital role in fostering collaboration and communication between sectors that do not always ‘speak the same language’. The Roadmap [43] covers a wide range of health and transportation research topics beyond physical activity, whereas the CHAT focused on research needs related to AT as an influence on health. We hope this commentary will help further disseminate all these materials, especially among health researchers who may not be as familiar with efforts concerning health-related research emerging from the transportation research community. Such efforts are addressing diverse issues interrelated with transportation infrastructure and transportation behavior including access, equity, AT, safety, pollution exposure and diverse environmental impacts of transportation related to public health.

## 3. The CHAT Program and Research Gaps in Active Transportation

The CHAT activities, including plenary addresses, lightning talks, conversations in breakout groups, and a dot-voting process [34], converged on four overarching research and implementation gaps concerning health and AT (Table 2). 

These areas included research on (1) Specific Populations, (2) Economics and Systems Analysis, (3) Evaluation of Emerging Activities, and (4) Messaging, Dissemination and Training. The conference agenda, speaker information and detailed summaries of each presentation are available in TRB E-Circular E-C264 [21].

**Figure 1:**
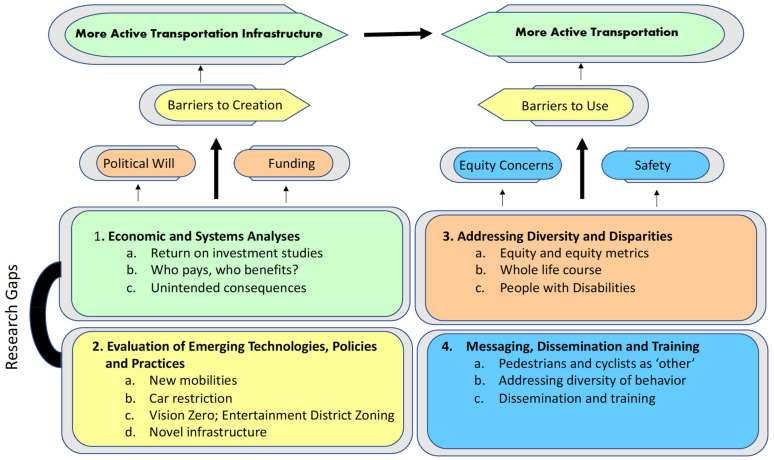
Research needs to increase transportation infrastructure and behavior.

Figure 1 summarizes interrelationships of these four themes with barriers to development and use of AT infrastructure. Conversations amongst conference attendees emphasized that better infrastructure alone does not lead to greater use of AT, resulting in the dual paths of (a) economic analyses and evaluation of innovative practices that give policy makers evidence concerning infrastructure and (b) calls for efforts to address equity of opportunity and messaging to overcome barriers to use. Attention to equity was a major feature of the entire workshop, with considerable discussion of transportation for under-represented and under-resourced communities, elderly and youth populations, and people with disabilities. Many of these discussions emphasized community-based approaches and an urgent need to collaborate with communities from the beginning of research projects to enhance the relevance of research results. 

Calls for economic and systems analysis approaches to transportation and health reflected the fact that the transportation and health sectors combined represent a complex system, with emergent properties and likelihood of unintended and unanticipated outcomes. Participants noted that economic analysis may help motivate advocates and policy makers to consider new approaches to transportation systems, emphasizing public and active transport, and highlighted the need for rigorous evaluation of natural experiments in innovative transportation approaches, because such innovations are rarely amenable to randomized trials. 

Transportation decision making is driven in large part by considerations of monetary costs and benefits. Monetizing the health impacts of changes to the built environment designed to support AT could help speed up implementation of AT interventions by demonstrating the costs and benefits of investment in AT and placing them on a common scale [46,47,48]. However, because different entities are responsible for transportation versus health care costs and benefits flow to different populations and organizations, such monetization might not stimulate more rapid investment in AT. Partnerships between transportation and public health might foster greater appreciation of health benefits associated with AT. Such benefits, most notably increased adherence to physical activity recommendations [2], as well as various costs associated with AT have been the subject of extensive research [49] and tools are available for their evaluation in specific programs [50]. These tools include health impact assessments that quantify the benefits of AT and indicate substantial net health benefits [51]. Although health impacts can be important in motivating an increased emphasis on AT, transportation professionals, not health professionals, have the expertise to design or renovate transportation infrastructure to support safe and comfortable AT. Collaboration between the transportation and health sectors is vital to accelerate adoption and evaluation of infrastructure and programs aimed at increasing AT.

Currently, several frameworks exist for health monetization, including, but not limited to, cost of illness (COI), cost-effectiveness or cost–benefit analysis, value of statistical life (VSL), real-world clinical outcomes, and health care costs [52,53]. Using these frameworks, the monetized direct and indirect benefits of health could be better integrated into larger economic models combining multiple sectors including health, transportation and employment [54]. In a few cases, such models suggest substantial benefits overall for investments in AT infrastructure that are not always captured in simpler models [55]. For example, a recent analysis of Prince Georges County, Maryland in the United States illustrates the use of the Integrated Transport and Health Impact Modeling (ITHIM) framework and a cost of illness/VSL approach and suggests significant health and monetary benefits of increasing active travel and reduced automobile use [48]. Both increased walking and increased biking resulted in benefits. 

However, existing methods for monetizing health costs and benefits in the context of the built environment have both strengths and weaknesses [54]. For example, the VSL approach can help monetize the exposure-related mortality changes induced by modifications of the built environment by capturing the societal value of lower mortality risk. However, it does not explicitly capture actual health expenditures that are incurred due to poor health. Additional theoretical and methodological research is needed to better justify application of different health economic frameworks in the context of AT. For example, cost-effectiveness analyses using quality adjusted life years can be promising in capturing both the intervention costs and benefits beyond changes in mortality. Interventions around AT have been shown to be cost effective in various contexts [56,57]. Lastly, there are challenges posed by a growing consensus of the need to plan for a more effective multi-modal transportation system in the US, instead of the traditional prominence given to motor vehicle travel and limited corridors for AT. 

Expanding infrastructure and improving safety for diverse users depends critically on understanding attitudes and the diversity of transportation behaviors and needs. Cross-cutting issues frequently mentioned included attention to intersections of AT with planning and housing sectors, more rigorous evaluation of natural experiments, and efforts to understand unintended consequences of policies and programs. Progress in rigorous evaluation and understanding unintended consequences was seen to depend on better data, coordinated and complementary funding from both the health and transportation sectors, and dissemination of evidence and training across disciplines—with special attention to trainees of underrepresented groups.

The themes of ethics, equity and empathy have been increasingly emphasized by policymakers, engineers and planners over the past decade, were highlighted in the closing plenary of the conference by Jennifer Toole. These themes were a major element of the conversations that occurred throughout the meeting [58]. These new elements should not be thought of as replacing the traditional three ‘E’s of traffic safety—education, engineering and enforcement, or a substitute for proposals to expand this model to seven ‘E’s by including economics, emergency response, enablement and ergonomics [59]. Rather these elements acknowledge that the transportation field needs to deliver on the promise of a safe, equitable, and sustainable transportation system that serves diverse populations with diverse transportation modes and needs. 

A focus on these elements of design for AT could help ameliorate some of the negative consequences of much current transportation planning and design for motor vehicle travel and its unintended environmental consequences. Attendees discussed the possibility that this change in emphasis could spark greater efforts to reconsider traditional narratives of efficiency and economics that have contributed to environmental injustice associated with transportation and planning decisions in the US and worldwide.

## 4. Innovative Practices, Building Strategic Institutional Relationships, and Cross-Cutting Needs

Several issues discussed in the CHAT Active Travel Behavior Research theme were mirrored in the Innovative Practices and Building Strategic Institutional Relationships themes. For example, the Innovative Practices breakouts emphasized the need for careful evaluation research and attention to unintended consequences, and the Strategic Relationships discussion brought up needs for policy implementation research [60] and greater attention to engaging diverse populations in planning, design and evaluation. Attention is also needed to tradeoffs and unintended consequences of innovation. For example, information and communication-based mobility-on-demand services (ride hailing, carsharing) may encourage multi-modal transportation by enhancing first- and last-mile connectivity. However, mobility-on-demand services may reduce AT by replacing utilitarian walking or bicycling trips and may be inaccessible to low-income people [61] and people with disabilities [62]. Increasing use of e-bikes and other new mobilities also warrants further attention. For e-bikes, levels of energy expenditure appear to reach near moderate intensity levels [63]. However, few studies have examined other physiological outcomes of e-cycling [64]. Nevertheless, e-bikes would appear to provide physical activity with lower levels of perceived exertion and higher speeds, possibly overcoming some barriers to uptake of cycling for active transportation [65]. 

Further issues arose among two or more CHAT themes. For example, there were recurring discussions of the need for better surveillance and data resources including more granular data concerning behavior and environments supportive of AT. Such data could contribute to evaluation of natural experiments as part of a program of developing ‘practice-based evidence’ to complement evidence generated from randomized trials of specific medications and other interventions [66]. Better data sharing between private sector transportation and technology companies and researchers could respond to this need and improve both surveillance and evaluation of natural experiments. For example, complications and barriers associated with using Google Street View images to extract environmental data were discussed at length [67]. Similar calls have been made at a variety of workshops addressing physical activity [40] and transportation [43]. Other recurring topics at the conference were examination of unintended and/or adverse consequences arising from poor planning, racism, or discrimination related to poverty [68], intersections of research needs concerning planning, land use and housing [45], greater attention to implementation and policy implementation research [60], and efforts at making research more accessible to practitioners. One topic that received little attention at the conference involved the potential interactions of autonomous vehicles and the prevalence of AT. A concern, and a topic for future conferences is that widespread use of autonomous vehicles could decrease AT because of the appeal of free, albeit sedentary time, made available by such vehicles [69]. Additionally, widespread use of such vehicles could increase both urban sprawl and congestion, decreasing walkability and making investment in transit less appealing [70]. Several reviews of the potential health impacts of autonomous vehicles have been published [71,72,73], with some emphasizing the role of local governments in determining the place of autonomous vehicles in US transportation networks [74]. Leaders of the Innovative Practices and Building Strategic Institutional Relationships themes are developing materials to further disseminate ideas and perspectives developed at the CHAT.

## 5. Active Transportation, Transit Equity and the COVID-19 Pandemic

The COVID-19 pandemic began a few months after the CHAT. The pandemic led to worldwide disruptions in transportation behaviors. It also resulted in significant changes in cycling, open streets policies, prevalence of pop-up bike lanes, pedestrianized streets and traffic volume. The declines in AT and specifically the inequities related to public transportation, due to real and perceived risks of COVID-19 and mitigation strategies, are of great interest as are some of the more positive changes in affordances for active leisure and transportation. Physical inactivity is associated with higher risk for severe COVID-19 outcomes [75] and in an ecological study found that sedentary travel levels were associated with increased COVID-19 hospitalizations [76], further reinforcing the importance of understanding AT in relation to the pandemic.

When the CDC issued social distancing recommendations, many employees teleworked through months of stay-at-home orders while millions of other individuals were deemed essential or frontline workers and continued to work outside the home. A significant proportion of these workers require public transportation to commute to work or undertake necessary errands (e.g., grocery shopping) and account for more than one-third of total transit commuters in the United States [77]. This finding is not unforeseen, as lack of a car is increasingly associated with poverty and essential and frontline workers have been designated as economically vulnerable because nearly half of these workers are employed in low-paid occupations (e.g., median wage of $15 dollars/hour or less) [20,78]. While 6% of White American households are carless overall, more than twice as many (14%) households of color are carless [79]. Carless households and public transit use are also disproportionately concentrated in major metropolitan areas, such as New York City, Chicago, and Washington, DC, which were among the hardest hit by COVID-19 morbidity, mortality and race/ethnic based disparities [20,80]. Many cities significantly reduced or suspended public transit service given the severity of the pandemic and in compliance with social distancing directives. These service disruptions negatively affected many individuals needing or working in transit service and widened existing health disparities and inequities by (1) increasing the risk of COVID-19 exposure for transit riders and/or operators; (2) jeopardizing the employment of transit operators; and (3) obstructing the ability of transit users to commute to and from their place of employment [19,24]. 

Despite negative effects of pandemic mitigation strategies on overall public transportation use and inequities related to ridership by essential workers, there are also positive lessons and opportunities arising that are important targets for further research, evaluation, and promotion. For example, Combs and Pardo (2021) created a database of over 1000 projects involving diverse interventions including partial street closures, reallocation of curb and non-curb space, bike share programs, and many others [81]. The database involves almost entirely urban areas (96%), and largely projects from North America (58%) and Europe (30%). 

A flurry of workshops, papers, and diverse efforts to understand the short- and long-term effects of the pandemic have begun [24,82]. Many unknowns will determine if use of public transportation rapidly returns to pre-pandemic levels and whether pandemic changes in public space for AT and associated increases in cycling can be preserved. Ongoing and improved surveillance of bicycle use and its environmental supports are needed to determine if these positive outcomes persist. The research needs identified in this 2019 conference will remain relevant, along with needs related to safety, transit equity, effectiveness of interventions aimed at overcoming the fear of infection, and investigations of how to improve transportation options and operations if the high levels of telecommuting observed during the pandemic are sustained in future years. 

The post-pandemic world is an opportunity for AT that requires us to integrate research challenges from before and after the pandemic [81]. This research should emphasize both positive and negative consequences of the pandemic for AT and seek to understand sustainability of positive environmental changes emerging worldwide as well as new directions for transit systems in an environment including greater prevalence of remote work.

## 6. Conclusions

The past decade has seen a distinct uptick in research interest on transportation and health and a growing body of research on AT, safety, and pollution effects on chronic disease. The prevalence of AT in the United States was stable or declining prior to the COVID pandemic (e.g., [8,83]) although increases are seen in some urban areas [8]. The resources described in this paper include a myriad of important research questions and rich guidance for investigators at all stages of their careers. In addition to research, the 2019 CHAT emphasized the vital need to accelerate uptake and evaluation of innovative practices to advance transportation and health goals. These include use of emerging technologies, better integration of diverse, longitudinal data streams, a focus on underserved populations and equity as well as efforts to preserve and expand positive changes in the transportation and leisure physical activity environments that occurred during the pandemic. Achieving these goals will involve greater efforts aimed at Building Strategic Institutional Relationships, the third theme of the CHAT. Attention to dissemination and implementation research and the emerging topic of policy implementation research could enhance efforts to develop interdisciplinary collaborations, along with attention to communication across sectors. All three of these domains should be addressed in an ethical, equitable and empathic framework. Both this conference and the 2015 TRB Moving to Higher Ground conference identified a wide variety of gaps in surveillance, practice and research. Systematic efforts to track whether these recommendations are adopted would be of value.

Greater attention to these themes could help overcome both barriers to development of AT infrastructure and barriers to its use (Figure 1). The CHAT research themes can contribute to the emerging conversation about how to adapt to the pandemic-related decline in the use of public transit that has now only partially recovered. Addressing this decline depends heavily on better communication and training related to risk perceptions and attitudes towards safety, but it also depends on rigorous evaluation of innovative practices. For example, many predict greater use of telework going forward. Proposals to emphasize transit patterns differing from the traditional focus on commuters going from suburb to downtown require careful evaluation and economic analyses. Generally, transportation agencies could be required to consider the health impacts of projects more comprehensively and to more comprehensively account for the equity impacts of decisions. Such consideration is likely to highlight benefits of AT projects and promote public and environmental health.

## Figures and Tables

**Table 1:** Activities related to research needs in transportation and health.

Activity	Scope	Citations
Transportation Research Board Moving Active Transportation to Higher Ground: Opportunities for Accelerating the Assessment of Health Impacts	“The main themes of the conference were policy and planning initiatives to increase active transportation, health impact assessments (HIAs), data collection and data analysis to quantify active transportation, and methods to forecast active transportation and its effects on health.”	Schneider et al. 2015 [32]
Arterial Roadways Research Needs and Concerns: Informing the Planning, Design, and Operation of Arterial Roadways Considering Public Health. Transportation Research Circular E-C239	“The purpose of the Task Force was to inform the planning, design, and operation of arterials considering public health.”	McAndrews et al. 2017 [36] TRB E-C239, 2018 [37] Christopher and McAndrews, 2018 [38]
Implementing Strategies to Enhance Public Health Surveillance of Physical Activity in the United States (2019)	“An ad hoc committee will be convened to develop strategies that support the implementation of recommended actions to improve national physical activity surveillance. Specifically, the committee will: convene a group of experts who will examine and build on existing recommended actions in four topical areas (children and youth, community supports for active transportation, health care, and workplaces) to identify specific strategies for implementing those recommended actions; … “	National Academies 2019 [39] Dunton et al. 2019 [40]
A Guidebook for Communications between Transportation and Public Health Communities	“The purpose of this study is to produce a user-friendly guidebook for state and local transportation professionals that identifies the challenges and best practices for successful communication and collaboration between transportation and public health professionals.”	Steedly et al. 2019 [26]
TRB Health and Transportation Subcommittee Centennial Paper	“The TRB Joint Subcommittee on Health and Transportation was created in 2011 to identify, advance and publish research and information to expand and improve current understanding and evaluation of the health impacts of federal, state, regional and local transportation policies, procedures and actions.”	Berrigan et al. 2019 [41]
National Cooperative Highway Research Program Research Report 932, “A Research Roadmap for Transportation and Public Health”.	“NCHRP Research Report 932 provides state departments of transportation, their transportation partners, and the public health community with a broad overview of highly relevant research needs at the intersection of transportation and public health in the United States.”	National Academies, 2019 [42] Dannenberg et al. 2021 [43]
Transportation Research Board 2019 Conference on Health and Active Transportation	“The purpose of this conference is to explore and collaborate on identifying impacts of the health effects of transportation policies, planning, and infrastructure, and to develop an understanding of the institutional opportunities and barriers for considering health within transportation field.”	Rodgers 2020 [21] This paper

**Table 2:** Research and implementation gaps identified in the December 2019 Conference on Health and Active Transportation.

Themes and Elements	Research Needs/Approaches	Key Collaborators
1. **Research on Specific Populations**		
Work with diverse populations including people of color and transit dependent people to address equity	Community-based research Combine approaches from transportation metrics and health disparities tools Develop Health equity performance metrics	Partners from under-resourced communities Researchers from geriatrics, active transport to school, disability and health disparities arenas.
b. Elderly		
c. Youth		
d. People with disabilities		
2. **Economic and Systems Analyses of Active Transportation**		
Return on investment studies	Greater multi-disciplinary collaboration Development, validation and dissemination of tools and methods Creative thinking about methods to address unintended consequences Development of joint transportation and health funding models	Transportation, health and ecological economists Systems modelers Social epidemiologists
b. Addressing who pays and who benefits		
c. Guides to health impacts and their economic consequences		
d. Unintended consequences		
3. **Emerging Technologies, Policies and Practices**		
New mobilities	Broad dissemination of experiences and results Evaluation of these “natural experiments”	Data scientists Policy researchers Dissemination and implementation research community
b. Car restrictions such as reduced parking availability		
c. Emerging data streams for surveillance and evaluation		
d. Accessible AT infrastructure investment		
e. Vision Zero		
f. Alcohol, pedestrians and cyclists; Entertainment District Zoning		
4. **Messaging, Dissemination and Training**		
Pedestrians and cyclists as ‘other’	Explorations of the social psychology of transportation Risk perception and fatalism research Development and support for short- and long-term interdisciplinary training in transportation and health	Communications researchers Public opinion researchers Behavioral psychologists Educators Transportation and public health programs and courses at colleges and universities Planners at transportation agencies and transit agencies Transportation Demand Management planning and engagement staff Community engagement staff
b. Culture and the diversity of behavior		
c. Dissemination of evidence and training across disciplines		
